# Risk factors for Veteran food insecurity: findings from a National US Department of Veterans Affairs Food Insecurity Screener

**DOI:** 10.1017/S1368980021004584

**Published:** 2022-04

**Authors:** Alicia J Cohen, David M Dosa, James L Rudolph, Christopher W Halladay, Michele Heisler, Kali S Thomas

**Affiliations:** 1 Center of Innovation in Long Term Services and Supports, VA Providence Healthcare System, Providence, RI, USA; 2 Department of Family Medicine, Alpert Medical School of Brown University, Providence, RI, USA; 3 Department of Health Services, Policy, and Practice, Brown University School of Public Health, Providence, RI 02903, USA; 4 Department of Medicine, Alpert Medical School of Brown University, Providence, RI, USA; 5 Center for Clinical Management Research, VA Ann Arbor Healthcare System, Ann Arbor, MI, USA; 6 Department of Internal Medicine, University of Michigan Medical School, Ann Arbor, Michigan; 7 Department of Health Behavior and Health Education, University of Michigan School of Public Health, Ann Arbor, MI, USA

**Keywords:** Food insecurity, Military Veterans, Social determinants of health, Military sexual trauma

## Abstract

**Objective:**

Food insecurity is associated with numerous adverse health outcomes. The US Veterans Health Administration (VHA) began universal food insecurity screening in 2017. This study examined prevalence and correlates of food insecurity among Veterans screened.

**Design:**

Retrospective cross-sectional study using VHA administrative data. Multivariable logistic regression models were estimated to identify sociodemographic and medical characteristics associated with a positive food insecurity screen.

**Setting:**

All US Veterans Administration (VA) medical centres (*n* 161).

**Participants:**

All Veterans were screened for food insecurity since screening initiation (July 2017–December 2018).

**Results:**

Of 3 304 702 Veterans screened for food insecurity, 44 298 were positive on their initial screen (1·3 % of men; 2·0 % of women). Food insecurity was associated with identifying as non-Hispanic Black or Hispanic. Veterans who were non-married/partnered, low-income Veterans without VA disability-related compensation and those with housing instability had higher odds of food insecurity, as did Veterans with a BMI < 18·5, diabetes, depression and post-traumatic stress disorder. Prior military sexual trauma (MST) was associated with food insecurity among both men and women. Women screening positive, however, were eight times more likely than men to have experienced MST (48·9 % *v*. 5·9 %).

**Conclusions:**

Food insecurity was associated with medical and trauma-related comorbidities as well as unmet social needs including housing instability. Additionally, Veterans of colour and women were at higher risk for food insecurity. Findings can inform development of tailored interventions to address food insecurity such as more frequent screening among high-risk populations, onsite support applying for federal food assistance programs and formal partnerships with community-based resources.

Food insecurity—defined as limited or uncertain availability of nutritionally adequate and safe food^(1)^—is associated with a range of adverse health outcomes^(2,3)^. Food insecurity is also associated with delayed or missed care^(4,5)^, increased acute care utilisation^(4,6,7)^ and higher healthcare costs^(6,8)^. In 2020, 10·5 % of US households—and 14·8 % of households with children—reported being food insecure at least some time during the prior 12 months^(9)^. This financial strain is often more pronounced in households with individuals with acute or chronic medical conditions^(10–12)^. Levels of food insecurity increased dramatically since the start of the COVID-19 pandemic, particularly among households with children and among racial and ethnic minority groups, due to a variety of factors including employer shut downs, loss of childcare and in-person schooling and health-related economic hardship^(9,13–15)^.

Limited research has focused on food insecurity among the 8 % of the US population that has served in the US armed forces. Many Veterans face unique economic and employment challenges following their military service, stemming both from service-related mental and physical health issues as well difficulty reintegrating into civilian life^(16)^. Previously published estimates of the prevalence of food insecurity among US Veterans vary from a low of 6 % to as high as 24 % depending on the criteria utilised^(3,16–20)^. Two studies have examined food insecurity among the general Veteran population using nationally representative survey data, with respective prevalence rates of 6·5 % in the prior 30 d^(17)^ and 8·4 % in the prior 12 months^(18)^. A study analysing data from 2002 to 2008 waves of the Veterans Aging Cohort Study, a multisite investigation of Veterans receiving Department of Veterans Affairs (VA) health care, found that 24 % of Veterans reported food insecurity^(3)^. Higher rates of food insecurity have been reported among Veterans of Iraq and Afghanistan (27 %)^(21)^, women Veterans (28 %)^(5)^, homeless and formerly homeless Veterans (49 %)^(22)^ and Veterans with serious mental illness (35 %)^(17)^.

The Veterans Health Administration (VHA), the country’s largest integrated federally funded health care system, has invested heavily in screening for and addressing Veteran food insecurity. Following 2015 congressional briefings on Veteran food insecurity, recommendations from a subsequently chartered VA Ensuring Veteran Food Security Workgroup, and a food insecurity screening pilot in VA homeless clinics^(22)^, VHA developed a national food insecurity screener in 2017. This food insecurity clinical reminder, which is integrated into the electronic health record, prompts staff to administer a single-item screening question annually to all Veterans receiving VA health care who are not residents of a long-term care facility. Veterans endorsing food insecurity based on the single-item screener are offered a referral to a social worker and/or a dietitian as part of the clinical reminder. The food insecurity clinical reminder was first piloted at six sites across the country in July 2017, and implemented across VA medical centres nationally in October 2017^(23)^.

Little is known about prevalence or correlates of food insecurity among Veterans receiving VHA care, as measured by this universal VA food insecurity screener. To address this gap, we analysed data from the first 18 months following implementation of the food insecurity clinical reminder to identify: (1) the prevalence of reported food insecurity and (2) selected sociodemographic, medical and psychosocial characteristics associated with a positive screen. Based on prior literature, we hypothesised that while many sociodemographic and medical characteristics associated with food insecurity among US Veterans would be similar to those in the general US population, US Veterans may have additional military service and/or trauma-related risk factors for food insecurity.

## Methods

### Data source and study cohort

Data were extracted from the VA Corporate Data Warehouse, a national data repository that includes VA administrative and electronic health record data. The cohort consisted of all Veterans who were screened using the VA food insecurity clinical reminder between when it was first piloted in July 1, 2017 and December 31, 2018. Veterans were eligible for screening with the clinical reminder if they (1) received VA clinical care during the above time period, and (2) were not residing in a long-term care facility. During this time period, 3 513 321 food insecurity clinical reminder screens were completed. An additional 7496 (0·2 %) of Veterans declined or were unable to answer the screening question, or were flagged in the electronic health record to receive the food insecurity clinical reminder but were ineligible due to residence in a long-term care facility. For those Veterans screened more than once during the study period, we restricted analyses to their first food insecurity screen, resulting in a final analytic sample of 3 304 702 Veterans.

### Measures

The food insecurity clinical reminder prompts VA clinical staff to ask each eligible Veteran: ‘In the past 3 months did you ever run out of food and were you not able to access more food or have the money to buy more food?’ (yes/no). A ‘yes’ response is considered a positive screen for food insecurity.

All clinical reminder data are housed in the Corporate Data Warehouse in a national ‘Health Factors’ database. Sociodemographic characteristics—including gender, age at the time of screening, race, ethnicity, marital status, history of military sexual trauma (MST) and Veteran enrolment priority status—were obtained from the medical record. MST is defined as sexual assault or harassment experienced during military service, and is a standardised screen administered to all Veterans. Enrolment priority determines Veterans’ eligibility for, and cost-share associated with, VA health benefits. We collapsed enrolment priority into three categories based on VA benefits: Veterans with some percentage of service-connected disability and disability-related VA compensation, Veterans not receiving VA disability compensation who are low income, and Veterans not receiving VA disability compensation who have income above the VA administered means test.

Most recent BMI was obtained from electronic health record vital sign data. We used the 10th revision of the International Classification of Diseases -10 codes to define medical and behavioural diagnoses including diabetes mellitus type II, hypertension, depression, post-traumatic stress disorder (PTSD) and substance use disorder. Separate VA clinical reminder data were used to define current tobacco use in the prior year. We defined homelessness/housing instability as having a positive homelessness clinical reminder screen and/or an International Classification of Diseases-10 code associated with homelessness or housing instability in the prior year. We identified Veterans living in a rural area using a standardised VA definition based on rural–urban commuting area codes.

### Statistical methods

Standard descriptive statistics (frequencies and percentages) were calculated for all variables. We estimated bivariate and multivariable logistic regression models to identify sociodemographic, medical and psychosocial characteristics associated with a positive food insecurity clinical reminder screen. Because of potential unobserved facility-level differences in screening practices and populations served, all models include fixed effects for VA medical centres. For covariates with ≥ 5 % missing values—race/ethnicity, marital status and BMI—we included missing as a separate category in the regression models. Missing values for the remaining variables were each ≤ 1 %. All analyses were conducted both for the full sample and stratified by gender.

In order to account for potential differences in screening practices during the initial ‘ramp up’ of the clinical reminder as sites were adapting to a new screening instrument, we conducted sensitivity analyses excluding the first 6 months of screening data and restricting our sample to Veterans screened between January 2018 and December 2018. R, version 3.6.1, was used for all analyses. This study was approved by the Providence VA Medical Center Institutional Review Board.

## Results

Between July 2017 and December 2018, 3 304 702 Veterans were screened using the food insecurity clinical reminder (Table 1). Of those screened, 3 005 797 (91·0 %) were male and 298 905 (9·0 %) were female. Female Veterans were overall younger than male Veterans. 43·6 % of women and 26·5 % of men identified as non-white. More than half of men (54·6 %) and nearly two-thirds of women (66·2 %) had some level of service-connected disability. Six percent of men and 8·2 % of women had experienced homelessness or housing instability during the prior year. Clinically, the majority of men (73·2 %) and women (69·9 %) were overweight or obese. Men were more likely than women to have a diagnosis of diabetes (25·7 % *v*. 11·4 %) and hypertension (54·6 % and 28·0 %), whereas women were more likely to have a diagnosis of depression (15·6 % *v*. 30·7 %) and PTSD (14·2 % *v*. 21·3 %). Nearly one-third of women (31·1 %) reported a history of MST, compared with 1·9 % of men.

**Table 1:** Characteristics of US Veterans Health Administration patients screened using the food insecurity clinical reminder, overall and by gender: July 2017–December 2018

Characteristics, *n* (%)	All Veterans (*n* 3 304 702)		Male Veterans (*n* 3 005 797)		Female Veterans (*n* 298 905)	
	*n*	%	*n*	%	*n*	%
Age						
18–34	327 916	9·9	262 203	8·7	65 713	22·0
35–44	309 411	9·4	247 201	8·2	62 210	20·8
45–54	414 583	12·5	350 519	11·7	64 064	21·4
55–64	616 436	18·7	546 335	18·2	70 101	23·5
≥ 65	1 636 356	49·5	1 599 539	53·2	36 817	12·3
Race						
White, Non-Hispanic	2 198 029	66·5	2 043 824	68·0	154 205	51·6
Black, non-Hispanic	573 391	17·4	483 020	16·1	90 371	30·2
Hispanic	216 034	6·5	192 561	6·4	23 473	7·9
Other, non-Hispanic	136 791	4·1	120 370	4·0	16 421	5·5
Missing	216 559	6·6	197 815	6·6	18 744	6·3
Marital status						
Married/partnered	1 749 026	52·9	1 643 620	54·7	105 406	35·3
Divorced/separated/widowed	949 157	28·7	835 055	27·8	114 102	38·2
Single, never married	441 903	13·4	374 089	12·4	67 814	22·7
Missing	164 616	5·0	153 033	5·1	11 583	3·9
Rural	1 106 532	33·5	1 031 236	34·3	72 296	25·2
Enrolment Priority*						
SC Disability†	1 838 974	55·6	1 641 146	54·6	197 828	66·2
Non-SC and low-income‡	735 168	22·2	677 176	22·5	57 992	19·4
Non-SC and not low-income§	719 751	21·8	678 616	22·6	41 135	13·8
Missing	10 809	0·3	8859	0·3	1950	0·7
Substance use disorder	299 230	9·1	279 239	9·3	19 991	6·7
Current smoker	796 003	24·1	734 826	24·4	61 177	20·5
Homelessness/housing instability	203 979	6·2	179 335	6·0	24 644	8·2
BMI						
< 18·5	26 442	0·8	23 219	0·8	3223	1·1
18·5–24·9	552 761	16·7	492 977	16·4	59 784	20·0
25–29·9	1 076 081	32·6	994 038	33·1	82 043	27·4
30–34·9	804 549	24·3	735 973	24·5	68 576	22·9
≥ 35	528 637	16·0	470 119	15·6	58 518	19·6
Missing	316 232	9·6	289 471	9·6	26 761	9·0
Diabetes	807 910	24·4	773 693	25·7	34 217	11·4
Hypertension	1 725 228	52·2	1 641 425	54·6	83 803	28·0
Depression	561 245	17·0	469 471	15·6	91 774	30·7
Post-traumatic stress disorder	489 391	14·8	425 617	14·2	63 774	21·3
History of military sexual trauma	148 677	4·5	55 736	1·9	92 941	31·1

Abbreviation: SC, service connected; VA, US Department of Veterans Affairs; BMI, body mass index.

* Enrolment priority determines US Veterans’ eligibility for, and cost-share associated with, VA health benefits.

† SC disability provides a monetary benefit paid to Veterans who are determined by VA to be disabled by an injury or illness that was incurred or aggravated during active military service.

‡ Non-service connected Veterans determined by the VA to be low-income.

§ Non-service connected Veterans who have income above the VA administered means test.

Overall, 44 298 Veterans (1·3 %) screened positive for food insecurity on their initial clinical reminder screen (1·3 % of men and 2·0 % of women, Table 2). There was substantial variation in the percentage of positive screens across VA medical facilities, ranging from 0·11 to 11·1 % (median 1·2 %, IQR 0·9–1·6 %). Compared with Veterans screening negative for food insecurity, Veterans with a positive screen were more likely to be < 65 years of age (77·9 % *v*. 50·2 %), and to identify as a racial/ethnic minority (46·3 % *v*. 27·7 %), non-married/partnered (70·2 % *v*. 41·7 %) and low income (44·1 % *v*. 21·9 %). Veterans with a positive food insecurity screen were also more likely have experienced homelessness or housing instability in the prior year (58·1 % *v*. 5·5 %); smoke tobacco (48·3 % *v*. 23·8 %); have a history of MST (11·6 % *v*. 4·4 %) and have a diagnosis of depression (36·2 % *v*. 16·7 %), PTSD (23·8 % *v*. 14·7 %) and/or substance use disorder (28·9 % *v*. 8·8 %). Trends were overall similar in gender-stratified analyses (Table 2). The prevalence of MST was substantially higher among women regardless of food insecurity status—48·9 % of women with a positive food insecurity screen and 30·7 % of women with a negative food insecurity screen reported a history of MST (compared with 6·0 % and 1·8 % of men, respectively).

**Table 2:** Characteristics of Veterans screened using the food insecurity clinical reminder, by screening status: July 2017–December 2018

Characteristics, *n* (%)	All Veterans				Male Veterans				Female Veterans			
	Positive screen (*n* 44 298)		Negative screen (*n* 3 260 404)		Positive screen (*n* 38 449)		Negative screen (*n* 2 967 348)		Positive screen (*n* 5849)		Negative screen (*n* 293 056)	
	*n*	%	*n*	%	*n*	%	*n*	%	*n*	%	*n*	%
Age												
18–34	6582	14·9	321 334	9·9	5186	13·5	257 017	8·7	1396	23·9	64 317	21·9
35–44	5885	13·3	303 526	9·3	4512	11·7	242 689	8·2	1373	23·5	60 837	20·8
45–54	7396	16·7	407 187	12·5	6103	15·9	344 416	11·6	1293	22·1	62 771	21·4
55–64	14 631	33·0	601 805	18·5	13 217	34·4	533 118	18·0	1414	24·2	68 687	23·4
≥ 65	9804	22·1	1 626 552	49·9	9431	24·5	1 590 108	53·6	373	6·4	36 444	12·4
Race												
White, non-Hispanic	21 769	49·1	2 176 260	66·7	19 278	50·1	2 024 546	68·2	2491	42·6	151 714	51·8
Black, non-Hispanic	14 567	32·9	558 824	17·1	12 215	31·8	470 805	15·9	2352	40·2	88 019	30·0
Hispanic	3898	8·8	212 136	6·5	3476	9·0	189 085	6·4	422	7·2	23 051	7·9
Other, non-Hispanic	2022	4·6	134 769	4·1	1700	4·4	118 670	4·0	322	5·5	16 099	5·5
Missing	2604	5·9	213 955	6·6	2258	5·9	195 557	6·6	346	5·9	18 398	6·3
Marital status												
Married/partnered	11 232	25·4	1 737 794	53·3	10 091	26·2	1 633 529	55·1	1141	19·5	104 265	35·6
Divorced/separated/widowed	20 111	45·4	929 046	28·5	17 215	44·8	817 840	27·6	2896	49·5	111 206	37·9
Single, never married	10 977	24·8	430 926	13·2	9353	24·3	364 736	12·3	1624	27·8	66 190	22·6
Missing	1978	4·5	162 638	5·0	1790	4·7	151 243	5·1	188	3·2	11 395	3·9
Rural	9936	22·4	1 096 596	33·6	8815	22·9	1 022 421	34·5	1121	19·2	74 175	25·3
Enrolment priority*												
SC disability†	20 559	46·4	1 818 415	55·8	17 143	44·6	1 624 003	54·7	3416	58·4	194 412	66·3
Non-SC and low-income‡	19 519	44·1	715 649	21·9	17 556	45·7	659 620	22·2	1963	33·6	56 029	19·1
Non-SC and not low-income§	3961	8·9	715 790	22·0	3547	9·2	675 069	22·7	414	7·1	40 721	13·9
Missing	259	0·6	10 550	0·3	203	0·5	8656	0·3	56	1·0	1894	0·6
Substance use disorder	12 809	28·9	286 421	8·8	11 710	30·5	267 529	9·0	1099	18·8	18 892	6·4
Current smoker	21 392	48·3	774 611	23·8	19 104	49·7	715 722	24·1	2288	39·1	58 889	20·1
Homelessness/housing instability	25 751	58·1	178 228	5·5	22 503	58·5	156 832	5·3	3248	55·5	21 396	7·3
BMI												
< 18·5	855	1·9	25 587	0·8	728	1·9	22 491	0·8	127	2·2	3096	1·1
18·5–24·9	11 226	25·3	541 535	16·6	9974	25·9	483 003	16·3	1252	21·4	58 532	20·0
25–29·9	12 594	28·4	1 063 487	32·6	11 183	29·1	982 855	33·1	1411	24·1	80 632	27·5
30–34·9	8311	18·8	796 238	24·4	7085	18·4	728 888	24·6	1226	21·0	67 350	23·0
≥ 35	6216	14·0	522 421	16·0	5029	13·1	465 090	15·7	1187	20·3	57 331	19·6
Missing	5096	11·5	311 136	9·5	4450	11·6	285 021	9·6	646	11·0	26 115	8·9
Diabetes	8935	20·2	798 975	24·5	8170	21·2	765 523	25·8	765	13·1	33 452	11·4
Hypertension	18 141	41·0	1 707 087	52·4	16 538	43·0	1 624 887	54·8	1603	27·4	82 200	28·0
Depression	16 027	36·2	545 218	16·7	13 254	34·5	456 217	15·4	2773	47·4	89 001	30·4
Post-traumatic stress disorder	10 547	23·8	478 844	14·7	8484	22·1	417 133	14·1	2063	35·3	61 711	21·1
History of military sexual trauma	5146	11·6	143 531	4·4	2288	6·0	53 448	1·8	2858	48·9	90 083	30·7

Abbreviations: SC, service connected; VA, US Department of Veterans Affairs; BMI, body mass index.

* Enrolment priority determines US Veterans’ eligibility for, and cost-share associated with, VA health benefits.

† SC disability provides a monetary benefit paid to Veterans who are determined by VA to be disabled by an injury or illness that was incurred or aggravated during active military service.

‡ Non-service connected Veterans determined by the VA to be low-income.

§ Non-service connected Veterans who have income above the VA administered means test.

Adjusted and unadjusted odd ratios for correlates of a positive food insecurity clinical reminder, stratified by gender, are presented in Table 3. In adjusted models specific to male Veterans, a positive food insecurity screen was associated with age < 65 years (18–34 years: adjusted OR (aOR) = 1·93, 95 % CI = 1·71, 2·18; 35–44 years: aOR = 1·87, 95 % CI = 1·68, 2·08; 45–54 years: aOR = 1·66, 95 % CI = 1·51, 1·82; and 55–64 years: aOR = 1·58, 95 % CI = 1·44, 1·73); identifying as non-Hispanic Black (aOR = 1·32, 95 % CI = 1·25, 1·40), Hispanic (aOR = 1·48, 95 % CI = 1·06, 2·08) or ‘other’ non-white, non-Hispanic race/ethnicity (aOR = 1·22, 95 % CI = 1·12, 1·33), being non-married/partnered (aOR = 1·36, 95 % CI = 1·30, 1·42), and having experienced homelessness or housing instability in the prior year (aOR = 13·79, 95 % CI = 12·36, 15·40). Low-income Veterans without service-connected disability compensation had higher odds of a positive food insecurity screen (aOR = 1·52, 95 % CI = 1·47, 1·58) relative to Veterans with service-connected disability compensation. Veterans who were clinically underweight (BMI < 18·5, aOR = 1·31, 95 % CI = 1·21, 1·41), had a diagnosis of diabetes (aOR = 1·13, 95 % CI = 1·09, 1·18), smoked tobacco (aOR = 1·35, 95 % CI = 1·30, 1·41), had a diagnosis of depression or PTSD (aOR = 1·25, 95 % CI, 1·20, 1·31 and aOR = 1·05, 95 % CI = 1·01, 1·09, respectively) or a history of MST (aOR = 1·42, 95 % CI = 1·35, 1·50) also had increased odds of a positive screen. History of substance use disorder was associated with a positive clinical reminder screen in bivariate analyses, but not in the adjusted model.

**Table 3:** Correlates of a positive food insecurity clinical reminder screen, by gender

	Male Veterans				Female Veterans			
Characteristics	OR	95 % CI	aOR	95 % CI	OR	95 % CI	aOR	95 % CI
Age								
18–34	1·64	1·55, 1·75	1·93	1·71, 2·18	1·13	1·05, 1·21	1·69	1·48, 1·92
35–44	1·50	1·41, 1·58	1·87	1·68, 2·08	1·17	1·10, 1·24	1·72	1·52, 1·95
45–54	1·44	1·36, 1·52	1·66	1·51, 1·82	1·03	0·96, 1·11	1·51	1·35, 1·69
55–64	2·40	2·24, 2·56	1·58	1·44, 1·73	1·04	0·97, 1·12	1·31	1·16, 1·48
≥ 65	ref		ref		ref		ref	
Race/ethnicity								
White, Non-Hispanic	ref		ref		ref		ref	
Black, Non-Hispanic	2·47	2·24, 2·72	1·32	1·25, 1·40	1·56	1·45, 1·69	1·24	1·16, 1·33
Hispanic	1·47	1·17, 1·85	1·48	1·06, 2·08	0·91	0·80, 1·03	1·00	0·89, 1·12
Other, Non-Hispanic	1·11	0·99, 1·25	1·22	1·12, 1·33	1·01	0·89, 1·15	1·14	1·00, 1·29
Missing	0·88	0·81, 0·96	1·07	0·96, 1·19	0·93	0·83, 1·05	1·21	1·07, 1·36
Marital Status								
Married/partnered	ref		ref		ref		ref	
Non-married/partnered	3·38	3·06, 3·73	1·36	1·30, 1·42	2·23	2·10, 2·37	1·36	1·27, 1·44
Missing	0·91	0·79, 1·05	1·27	1·10, 1·46	0·81	0·69, 0·94	1·26	1·08, 1·47
Rural	0·57	0·50, 0·65	1·02	0·91, 1·15	0·70	0·63, 0·78	0·99	0·91, 1·09
Enrolment priority status*								
SC Disability†	ref		ref		ref		ref	
Non-SC and low-income‡	2·96	2·80, 3·13	1·52	1·47, 1·58	2·15	2·02, 2·29	1·52	1·43, 1·62
Non-SC and not low-income§	0·35	0·31, 0·38	0·85	0·79, 0·91	0·47	0·42, 0·53	0·93	0·83, 1·05
Substance use disorder	4·42	4·10, 4·77	0·97	0·93, 1·02	3·35	3·11, 3·62	1·05	0·96, 1·14
Current smoker	3·11	2·87, 3·38	1·35	1·30, 1·41	2·56	2·40, 2·74	1·53	1·42, 1·63
Homelessness/housing instability	25·23	22·06, 28·85	13·79	12·36, 15·40	15·82	14·58, 17·16	10·70	9·77, 11·70
BMI								
<18·5	2·52	2·31, 2·75	1·31	1·21, 1·41	2·08	1·75, 2·47	1·53	1·26, 1·87
18·5–24·9	ref		ref		ref		ref	
25–29·9	0·83	0·80, 0·86	0·73	0·71, 0·76	0·84	0·79, 0·89	0·86	0·80, 0·94
30–34·9	0·69	0·67, 0·72	0·67	0·64, 0·69	0·89	0·83, 0·96	0·89	0·81, 0·98
≥ 35	0·81	0·77, 0·85	0·71	0·67, 0·74	1·05	0·98, 1·12	0·96	0·88, 1·05
Missing	1·22	1·06, 1·41	0·90	0·83, 0·98	1·25	1·09, 1·43	1·07	0·96, 1·20
Diabetes	0·78	0·73, 0·83	1·13	1·09, 1·18	1·17	1·08, 1·27	1·22	1·11, 1·33
Hypertension	0·63	0·58, 0·67	0·84	0·80, 0·88	0·97	0·90, 1·04	0·93	0·87, 1·00
Depression	2·90	2·69, 3·12	1·25	1·20, 1·31	2·07	1·96, 2·19	1·22	1·15, 1·30
Post-traumatic stress disorder	1·73	1·62, 1·85	1·05	1·01, 1·09	2·05	1·92, 2·19	1·05	0·99, 1·13
History of military sexual trauma	3·44	3·19, 3·71	1·42	1·35, 1·50	2·15	2·01, 2·29	1·49	1·40, 1·59

Abbreviations: aOR, adjusted OR; SC, service connected; VA, US Department of Veterans Affairs, BMI, body mass index.

Multivariable logistic regression model, adjusted for VA Medical Center-level fixed effects.

* Enrolment priority determines Veterans’ eligibility for, and cost-share associated with, VA health benefits.

† SC disability provides a monetary benefit paid to Veterans who are determined by VA to be disabled by an injury or illness that was incurred or aggravated during active military service.

‡ Non-service connected Veterans determined by the VA to be low-income.

§ Non-service connected Veterans who have income above the VA administered means test.

Correlates of a positive clinical reminder were generally similar for women, with the exception that identifying as Hispanic was not associated with higher odds of a positive food insecurity (aOR = 1·00, 95 % CI = 0·89, 1·12). Additionally, while PTSD among women trended towards higher odds of a positive screen in adjusted analyses, this did not reach statistical significance (aOR = 1·05, 95 % CI = 0·99, 1·13) (Table 3). Adjusted and unadjusted odd ratios for correlates of a positive food insecurity clinical reminder for the overall cohort were similar to correlates among male Veterans given that 91 % of the cohort was male (see online supplementary material, Supplemental Table 1).

Sensitivity analyses excluding a 6-month ‘ramp-up’ period from July 2017 to December 2017 did not yield substantively different findings relative to results from analyses using the full July 2017–December 2018 period.

## Discussion

This study is, to our knowledge, the first to examine prevalence and risk factors associated with US Veteran food insecurity as identified in a nationwide VA food insecurity screener. More than 3·3 million non-institutionalised Veterans were screened for food insecurity in the first 18 months following implementation of the VA food insecurity clinical reminder. More than 44 000 Veterans were identified as food insecure on their initial screen, representing 1·3 % of men and 2·0 % of women. While measures of lower income and financial hardship were associated with a positive food insecurity clinical reminder screen, a positive screen was also associated with numerous medical and trauma-related comorbidities such as MST. Veterans of colour and women were also at increased risk for a positive food insecurity screen.

The prevalence of Veteran food insecurity identified by the VA food insecurity clinical reminder is markedly lower than rates found in prior studies^(3,5,17–19)^. This may reflect differences in the single-item question used in the VA screener compared with those used in prior research^(3,5,17,18,21)^. For example, the gold standard USDA Food Security Survey Module includes questions such as being ‘worried’ whether food would run out before there was money to buy more^(24)^. In contrast, the VA food insecurity clinical reminder asks whether a Veteran has ‘run out of food,’ a more severe form of food hardship. Additionally, the food insecurity clinical reminder provides binary ‘yes/no’ response options, which have been shown to identify substantially less need than offering ‘often/sometimes/never’ categories^(25)^. For these reasons, it is likely that the VA screener was capturing only the most severe cases of food insecurity among Veterans. Reported rates of food insecurity may also have been lower in our sample compared with survey-based estimates due to well-recognised barriers to disclosure of food insecurity in clinical settings including stigma, fear of judgment and concern about being reported to child protective services^(26–29)^. As with other food insecurity instruments commonly used both in clinical settings and in survey research^(24,30)^, the VA clinical reminder also focuses on financial barriers to food access and does not provide information on the nutritional adequacy of the food Veterans are able to obtain.

Similar to prior studies, we found that Veterans who were non-married/partnered^(16,17,20,21)^, low income^(3,5,16,17,20)^ or experienced housing instability within the past year^(3,22)^ were at increased risk for food insecurity. While the association between food insecurity and other markers of financial hardship such being low income or having unstable housing is unsurprising, findings of increased risk for food insecurity among those who are non-married/partnered may reflect having fewer financial reserves and potentially less social support to help stretch limited resources. Also consistent with other studies, we found that Veterans of colour were at increased risk of food insecurity^(3,17)^. This increased risk likely reflects a number of complex and interrelated factors including a disproportionate burden of poverty and unemployment resulting from structural racism and disparities in social and economic opportunity^(14,31)^, as well as experiences of racism and racial and ethnic discrimination^(32,33)^.

Also consistent with prior studies, we found that Veterans with a history of depression and/or PTSD had higher odds of a positive food insecurity screen^(3,5,17,20)^. Veterans with diabetes also had higher odds of food insecurity, which can impede pharmaceutical-based efforts to ensure adequate control of this diet-related disease. This is consistent with prior work finding poorer diabetes control among both Veterans and non-Veterans experiencing food insecurity^(3,10,34)^. Although US households in rural areas experience food insecurity at rates significantly higher than the national average^(9)^, we did not find an association between living in a rural area and food insecurity. One reason for this may be that because the clinical reminder question focuses on financial barriers to obtaining food, the screener does not explicitly assess other barriers to food access that may be more salient in rural areas such as limited food availability or lack of transportation.

Findings from our study and others^(17,20,21)^ that Veterans under 65 were more likely to screen positive for food insecurity likely reflects multiple factors. Volunteer-era (i.e. post-Vietnam) Veterans have both poorer financial stability and higher levels of material hardship than their counterparts from earlier service periods^(17,18,21,35)^. Volunteer-era Veterans are more likely to come from a lower socioeconomic background and have lower educational attainment^(36,37)^, and are more likely to report a history of childhood or other trauma prior to military service^(38)^. Each of these factors has been associated with higher risk of food insecurity later in life^(39,40)^. Furthermore, Veterans age 65 and older may be more likely to have resources to promote financial stability compared to Veterans under age 65. These resources may include social security retirement benefits, receipt of a military pension or retirement savings from a civilian job.

Veterans with some degree of service-connected disability—and who therefore also receive VA disability-related compensation—had lower odds of a positive food insecurity screen than low-income Veterans without a service-connected disability. This parallels findings of Montgomery and colleagues pertaining to service-connected disability and Veterans’ risk of homelessness and housing instability^(41)^. Together, these findings suggest that VA disability compensation provides some measure of protection from material hardship. Future work is needed to better understand how this protective effect may vary by type and severity of service-connected disability as well as benefit amount. Findings also suggest that Veterans who are low-income and not receiving VA disability-related compensation may benefit from additional targeted food insecurity screening efforts.

Our study is the first to have specifically examined the association between MST and food insecurity. There is, however, a well-established association between food insecurity and past and/or current trauma such as sexual or physical violence^(42)^ as well as history of adverse childhood experiences^(40)^. Prior studies have also found an association between history of MST and post-deployment homelessness^(43,44)^. These relationships are likely multifactorial and reflect the association between trauma and medical and mental health-related comorbidities, as well as higher rates of unemployment, financial hardship and decreased self-management capacity. Similar to our findings that history of MST was independently associated with food insecurity, Brignone and colleagues found that MST was independently associated with homelessness even after adjusting for co-occurring mental health conditions^(44)^. Taken together, these findings underscore the complex interplay between trauma exposure and subsequent material hardship.

History of MST was associated an increased odds of food insecurity among both men and women. Woman overall, however, were nearly 17 times more likely than men to have experienced MST (31·1 % of women *v*. 1·9 % of men), which is consistent with previously reported rates of MST^(45)^. Among women screening positive for food insecurity, nearly half (48·9 %) reported a history of MST. Compared with men, women overall also had twice the rate of depression, 50 % higher rates of PTSD and as in prior studies they were also more likely to have experienced recent homelessness or housing instability^(41,46)^, all of which were associated with increased risk for food insecurity. Each of these factors highlights the unique and complex challenges commonly faced by women Veterans.

Collectively, these findings can help clinicians focus additional targeted screening efforts which may include both more frequent screening among populations at particularly high risk for food insecurity, and also expansion of routine screening beyond primary care to include settings such as mental health clinics. Identifying Veterans vulnerable to food insecurity is particularly urgent given the current COVID-19 pandemic, which has both exacerbated existing disparities around food access and plunged many who were previously food secure into new material hardship^(15)^. In some settings such as VA homeless clinics, it may be appropriate to screen for food insecurity at every visit. The strong association independent between food insecurity and both MST and PTSD underscores the need for trauma informed care regardless of screening setting.

Our findings can also help providers and health care organisations prioritise the development of wrap-around, team-based interventions tailored to specific high-risk groups. Given that Veterans with diabetes and mental health-related comorbidities are at increased risk for food insecurity, targeted trainings regarding risks for and sequalae of food insecurity may be indicated for those providing clinical care for these populations including primary care and mental health providers, endocrinologists, pharmacists and dietitians. Trainings should cover the need to review medication lists with patients for any cost-related barriers to adherence, medications with high risk for hypoglycaemia or medications requiring specific food availability, as well as the importance of providing context-appropriate nutritional counselling based on patients’ medical and social circumstances. Interventions to ameliorate food insecurity may include development and/or expansion of onsite food pantries, referrals to community-based emergency food resources to meet immediate needs, onsite support applying for federal food assistance programs and/or direct provision of food through produce prescriptions or medically tailored meal programs. VA facilities may also partner with Veteran Service Organizations to facilitate connecting Veterans with available community and governmental resources.

The substantial variation in rates of positive screens across individual VA medical facilities likely reflects several factors including both geographical variation in the community-level prevalence of food insecurity as well as facility-level differences in screening practices and populations of Veterans served. Although the VA food insecurity clinical reminder question and follow-up prompts are uniform across VA medical centres, there is local variation in who administers the screening (e.g. clinician, nurse, medical assistant, social worker, dietitian) and whether the screener is administered prior to seeing a provider or during the clinical encounter. Additional research is needed to explore variation in how—and how reliably—screening is administered, as well as how variation in screening administration may impact Veterans’ responses. Prior work has found patients prefer self-administered paper or tablet-based food insecurity screening rather than being screened verbally^(26,47,48)^, and that disclosure rates for food insecurity in clinical settings are higher when screening is self-administered^(47–49)^. While the VA food insecurity clinical reminder—similar to other VA clinical reminders—is currently designed to be verbally administered by a member of the clinical care team, considering potential mechanisms for self-administered screening may be warranted.

Future work should examine variation at both the individual and medical centre level in how Veterans’ needs are addressed once they are identified as food insecure. There is also a need to better understand how Veterans’ experiences of food insecurity and food insecurity clinical reminder screening results may change over time, as well as optimal intervals for rescreening and whether this should vary by population. Finally, in April of 2021, which post-dated our study period, VHA updated the food insecurity clinical reminder to use a two-question instrument that has been validated to assess risk for food insecurity in clinical settings^(30)^. Future work should explore how the prevalence of food insecurity identified within VHA may change with this new instrument.

### Limitations

Our study has several limitations. First, because the VA food insecurity clinical reminder asks about particularly severe food hardship it may be underestimating the true prevalence of food insecurity among Veterans receiving VA care. While future validation studies are needed to understand how findings from the VA screener compare with other food insecurity instruments, it seems likely that those Veterans identified by the VA clinical reminder are at particularly high risk for having immediate food needs. Second, we were only able to analyse data for Veterans who presented for care within VHA and were screened during the first 18 months following implementation of the food insecurity clinical reminder. In particular, we were unable to examine food security status for those Veterans who did not engage in VHA care during the 18-month study period either because they did not seek healthcare during this period, they sought care outside of the VA using other benefits such as Medicare or Medicaid and/or they sought care in the community through the Choice Act^(50)^. A unique strength of this study is we were able to use a national administrative VA database to evaluate the entire population of 3·3 million Veterans screened. Third, there is local variation in how screening is administered which could impact Veterans’ response. We did, however, include VA medical centre-level fixed effects in our models to account for stable facility-level differences in screening practices and populations. Fourth, responses may have been influenced by perceived stigma, social-desirability bias and Veterans’ comfort with or trust in the person administering the screening. Results may have varied with a self-administered screener^(49)^.

## Conclusions

Systematic universal screening for food insecurity in the VA is a critical first step towards identifying Veterans currently experiencing or at high risk for experiencing food insecurity. Future work is needed to identify best practices for connecting Veterans experiencing food insecurity with VA and community resources to most effectively address the unique needs of the Veteran population.

## Supplementary material

For supplementary material accompanying this paper visit https://doi.org/10.1017/S1368980021004584. click here to view supplementary material
