# Supplementary material for: Risk factors for Veteran food insecurity: findings from a National US Department of Veterans Affairs Food Insecurity Screener
Source: Public Health Nutr. 2021 Nov 8;25(4):819–28. doi: 10.1017/S1368980021004584 (PMC8957505; doi:10.1017/S1368980021004584)
Supplement: Supplementary file 1 [file S1368980021004584sup001.docx]

| Supplement Table 1: Correlates of a Positive Food Insecurity Clinical Reminder Screen, Overall Cohort | | | |
| --- | --- | --- | --- |
| Characteristics | OR (95% CI) | aOR (95% CI) | |
| Gender | | | |
| Male | ref | 1.00 (ref) | |
| Female | 1.54 (1.44, 1.65) | 0.92 (0.88, 0.97) | |
| Age | | | |
| 18-34 | 1.60 (1.51, 1.69) | 1.90 (1.69, 2.13) | |
| 35-44 | 1.49 (1.42, 1.58) | 1.87 (1.69, 2.07) | |
| 45-54 | 1.40 (1.33, 1.48) | 1.66 (1.52, 1.83) | |
| 55-64 | 2.18 (2.05, 2.32) | 1.57 (1.44, 1.71) | |
| ≥65 | ref | ref | |
| Race/ethnicity | | | |
| White, non-Hispanic | ref | ref | |
| Black, non-Hispanic | 2.37 (2.17, 2.59) | 1.31 (1.24, 1.39) | |
| Hispanic | 1.39 (1.13, 1.71) | 1.41 (1.04, 1.92) | |
| Other, Non-Hispanic | 1.11 (0.99, 1.25) | 1.21 (1.11, 1.32) | |
| Missing | 0.88 (0.82, 0.95) | 1.08 (0.98, 1.20) | |
| Marital Status | | | |
| Married/partnered | ref | ref | |
| Non-married/partnered | 3.29 (3.01, 3.61) | 1.36 (1.30, 1.43) | |
| Missing | 0.89 (0.78, 1.01) | 1.27 (1.12, 1.45) | |
| Rural | 0.57 (0.50, 0.65) | 1.01 (0.91, 1.13) | |
| Enrollment Priority Status^a^ | | | |
| SC Disability^b^ | ref | ref | |
| Non-SC and low-income^c^ | 2.82 (2.68, 2.96) | 1.53 (1.48, 1.58) | |
| Non-SC and not low-income^d^ | 0.35 (0.32, 0.38) | 0.85 (0.80, 0.91) | |
| SUD | 4.22 (3.95, 4.52) | 0.98 (0.94, 1.03) | |
| Current Smoker | 3.00 (2.79, 3.23) | 1.38 (1.32, 1.44) | |
| Homelessness/Housing Instability | 23.96 (21.24, 27.02) | 13.28 (12.06, 14.62) | |
| BMI | | | |
| <18.5 | 2.48 (2.29, 2.69) | | 1.33 (1.23, 1.43) |
| 18.5-24.9 | ref | | ref |
| 25-29.9 | 0.82 (0.79, 0.85) | | 0.75 (0.73, 0.77) |
| 30-34.9 | 0.72 (0.69, 0.74) | | 0.69 (0.67, 0.72) |
| ≥35 | 0.86 (0.82, 0.89) | | 0.74 (0.71, 0.78) |
| Missing | 1.22 (1.06, 1.40) | | 0.92 (0.85, 1.00) |
| Diabetes | 0.78 (0.74, 0.83) | | 1.14 (1.10, 1.18) |
| Hypertension | 0.63 (0.59, 0.67) | | 0.85 (0.81, 0.89) |
| Depression | 2.83 (2.63, 3.04) | | 1.25 (1.20, 1.30) |
| PTSD | 1.82 (1.70, 1.94) | | 1.05 (1.02, 1.09) |
| History of MST | 2.85 (2.67, 3.04) | | 1.44 (1.38, 1.50) |

^a^Enrollment priority determines Veterans’ eligibility for, and cost-share associated with, VA health benefits.

^b^Service-connected (SC) disability provides “a monetary benefit paid to Veterans who are determined by VA to be disabled by an injury or illness that was incurred or aggravated during active military service.”^50^

^c^Non-service connected Veterans determined by the VA to be low-income

^d^Non-service connected Veterans who have income above the VA administered means test

Multivariable logistic regression model, adjusted for VA Medical Center-level fixed effects

Abbreviations: SC, Service Connected; SUD, Substance Use Disorder; MST, Military Sexual Trauma; PTSD, Posttraumatic stress disorder
